# Vomiting in newborns as a result of a duodenal membrane: two case reports

**DOI:** 10.31744/einstein_journal/2020RC4641

**Published:** 2020-11-18

**Authors:** Gabriela Bonente Herculano de Andrade, Barbara Said Marin, Daniela Nasu Monteiro Medeiros, Mauricio Gustavo Ieiri Yamanari, Eduardo Juan Troster

**Affiliations:** 1 Hospital Israelita Albert Einstein São PauloSP Brazil Hospital Israelita Albert Einstein, São Paulo, SP, Brazil.; 2 Hospital Israelita Albert Einstein Faculdade Israelita de Ciências da Saúde Albert Einstein São PauloSP Brazil Faculdade Israelita de Ciências da Saúde Albert Einstein, Hospital Israelita Albert Einstein, São Paulo, SP, Brazil.

**Keywords:** Duodenal membrane, Gastroesophageal reflux, Malnutrition, Child

## Abstract

Vomiting episodes in newborns are extremely common and often attributed to gastroesophageal reflux. The symptoms of vomiting, however, may be caused by other complications. In this report, we present two cases of a 1-month-old male and a 2-month-old female, both presenting vomiting episodes that led to malnutrition. Some pediatricians often attribute the diagnosis of gastroesophageal reflux to newborns that are vomiting; however, there is a portion of the population that has other causes that lead to similar symptoms. The pediatrician should be alert to the clinical signs of weight loss, dehydration and malnutrition to investigate other causes of vomiting.

## INTRODUCTION

Vomiting episodes in newborns are extremely common and often attributed to gastroesophageal reflux (GER), a physiologic process caused by the passage of gastric contents into the esophagus.^(^^1^^)^ Vomiting, however, is sporadic and may be caused by other complications. Most inborn errors of metabolism appear early in the newborn period, with vomiting and failure to thrive.^(^^2^^)^

It is important to evaluate when a case of vomiting is leading to a significant effect on the newborn health and nutritional status. The occurrence of vomiting with rapid weight loss, in the first weeks of life, should raise the hypothesis of anatomical abnormality of the digestive tract. Polyhydramnios in prenatal care, detected on morphological ultrasound, may be indicative of fetal intestinal obstruction.^(^^1^^)^

In this report, the authors bring the cases of a 1-month-old male and a 2-month-old female, both presenting vomiting episodes that led to malnutrition. More detailed, it was thought that it could be GER. After a deeper examination, the differential diagnosis led to a duodenal membrane. The case of the male patient required surgical intervention.

## CASE REPORT

### Case 1

A 1-month-old male infant was brought to our organization institution because of recurrent vomiting since birth and apparent malnutrition. The patient presented with bad general condition, with very low weight. Bilious vomiting was the only symptom, but his crying indicated severe pain.

A few days earlier, the patient was taken to another hospital, where his excessive weight loss and vomiting episodes were reported. Initially, the diagnosis was GER. It was decided that he would be breastfed more frequently, and complemented with formula. This proved to be a bad choice, since the vomiting episodes increased. The abdomen was distended and tender. Hypertrophic pyloric stenosis was suggested as a differential diagnosis.

**Figure 1:**
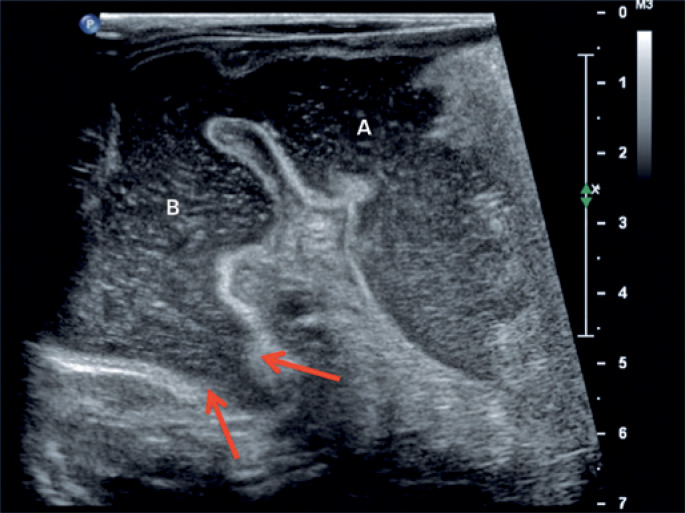
Ultrasonography with a high frequency linear transducer shows distension of the gastric antrum (A) and duodenal bulb (B). The duodenal membrane reduces the lumen of the second duodenal portion (arrows)

**Figure 2:**
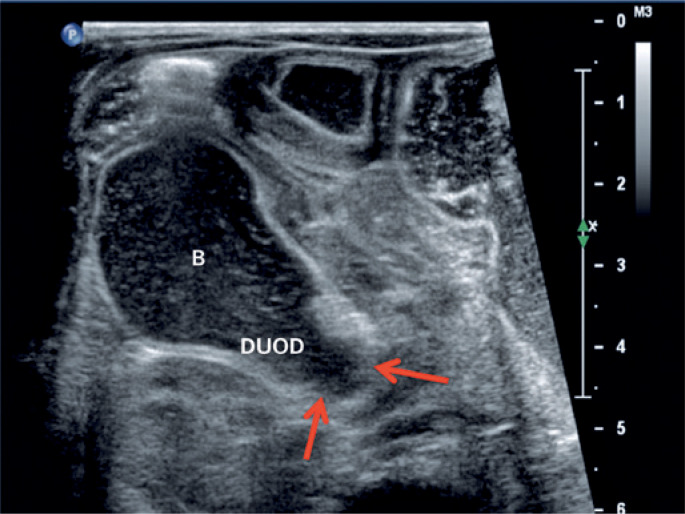
Ultrasonography with a high frequency linear transducer shows distension of the duodenal bulb, (B) with abrupt tapering in the second duodenal portion, corresponding to the local duodenal membrane

**Figure 3:**
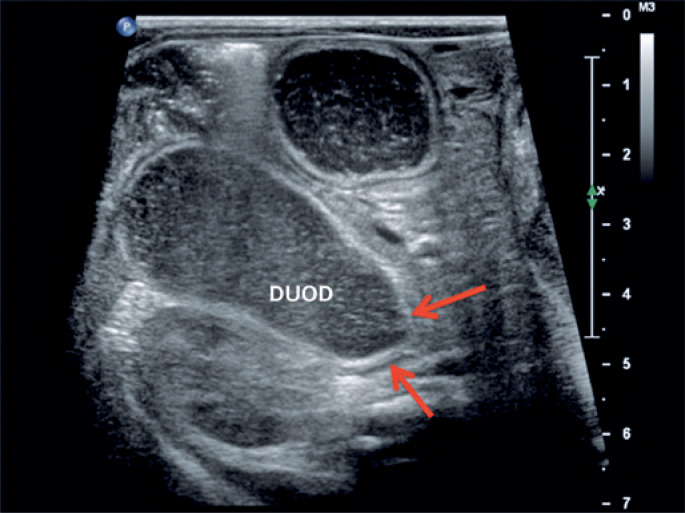
Ultrasonography with a high frequency linear transducer shows distension of the duodenal bulb with abrupt tapering (arrows) in the second duodenal portion, corresponding to the local duodenal membrane

At our hospital, an abdominal ultrasound (US) was performed ( Figures 1 to 3 ), which excluded pyloric stenosis or other lesions in the abdomen. A duodenal membrane was detected. There was an obstruction at the second portion of the duodenum with upstream dilatation.

The child was very malnourished and had to be admitted to the intensive care unit (ICU). Later, he underwent abdominal surgery. Total excision of the membrane was performed. Postoperative period went uneventful, except for a perineal candidiasis due to use of antibiotics. The infant was discharged 20 days postoperatively. He remains well after 1 year of follow-up.

### Case 2

A 2-month-old female infant admitted to the Pediatric ICU of the *Hospital Municipal Dr. Moysés Deutsch* , São Paulo (SP), Brazil, for 28 days, with a history of being born at term, Apgar score of 9/10, birth weight 3,260g, without complications during pregnancy. She had difficulty to nurse and frequent bilious vomits since birth, with no weight gain and neonatal jaundice (total bilirubin of 17) with phototherapy for 1 day. Since her weight loss progressed and vomiting episodes worsened, she was admitted to the hospital presenting dehydration and laboratory tests showing hypochloremic metabolic alkalosis and hyponatremia. After clinical stabilization, pediatric surgery opted for exploratory laparotomy, and diagnosis was made during surgery. The findings were: duodenal Ladd's bands, intestinal rotation, duodenal membrane obstructing the lumen of the organ. These findings were not seen in the ultrasound examination. During surgery, lysis of the Ladd's bands, duodenoduodenal anastomosis, and tactic appendectomy were performed due to obstruction second duodenal portion.

In the postoperative period, the infant presented vomiting and persistent hypoglycemia (dextro and serum glucose differing at the time of hypoglycemia), requiring high concentrations of glucose and hydrocortisone (25mg/m^2^). At the third and eighth postoperative days, the patient presented evisceration, and the intestinal loops were reintroduced in the abdomen. Since the clinical picture deteriorated, antibiotic therapy (vancomycin and meropenen) was extended. In the 11^th^ postoperative day, she evolved with fungal septic shock (hemoculture of the peripheral insertion central catheter - PICC with growth of *Candida albicans* ) and required vasoactive drugs and mechanical ventilation. Two days later, the patient's condition improved and she was extubated. She received a transfusion of red blood cell concentrate, and evolved with palpebral edema, crackles, dyspnea, had to be reintubated due to worse bilateral radiologic images, acute transfusion-related lung injury (TRALI) as the probable cause.

The patient was discharged after 3 months of hospitalization.

## DISCUSSION

Congenital duodenal obstruction is a common cause of intestinal obstruction in newborns.^(^^3^^)^ This defect is caused by an alteration in the canalization of the duodenum, which occurs between the eighth and tenth weeks of gestation. Congenital duodenal obstruction affects approximately one in 2,500 to 10,000 live births (almost half of all neonatal intestinal obstruction cases). Other studies showed the incidence is roughly one in 5,000 to 10,000 live births, and nearly 50% are associated with other malformations.^(^^4^^-^^6^^)^ The differential diagnoses of vomiting in newborns are infection, malformations and metabolic diseases.

The mortality rate was 6% to 58% of cases, but in recent studies, the initial postoperative survival rate has improved from 60% to 90%. Over 50% of children have other anomalies, such as cardiopathies (20%), malrotation (20%), Down syndrome (30%) and others. The survival of neonates has improved because of advances in surgical management, intensive care medicine, and postoperative nutritional support. ^(^^3^^,^^7^^)^ Our male patient had no other malformation.

These obstructions can be classified as complete or partial, and the causes are classified as intrinsic (duodenal atresia and membrane) or extrinsic (malrotation with Ladd's band and annular pancreas). They are also classified according to the types of duodenal membrane: intact membrane causing total obstruction, and perforated membrane leading to partial obstruction (2% of cases). The membrane is usually located in the second portion of the duodenum, near the ampulla of Vater.^(^^7^^-^^9^^)^

There are many clinical manifestations related to the type of obstruction, such as nausea, vomiting and weight loss. When the child has a partial obstruction, it is difficult to make diagnosis. In the case of the male patient, his stools were not acolic, suggesting partial obstruction. When the diagnosis is made late, the patient may evolve with malnutrition, dehydration and hydroelectrolytic disorder. One study showed the most common features were vomiting (100% of cases – non-bilious in 19%, and bilious in 81%), epigastric fullness in 70%, dehydration in 22% of neonates. In severe cases, shock is present in almost 19% of children. This study showed 37% of neonates had symptoms after 10 days of life.^(^^7^^-^^9^^)^

There is a possibility of prenatal diagnosis, and endoscopic intervention could be an option.^(^^8^^)^

Pediatricians must consider differential diagnoses in case of vomiting, especially when the patient fails to thrive, as reported in these cases.

## CONCLUSION

Some pediatricians often attribute the diagnosis of gastroesophageal reflux to newborns that are vomiting; however, there is a percentage of the population that has other causes that lead to similar symptoms. Pediatricians should be alert to the clinical signs of weight loss, dehydration and malnutrition to investigate other causes of vomiting.
