# Supplementary material for: Vomiting in newborns as a result of a duodenal membrane: two case reports
Source: Einstein (Sao Paulo). 2020 Nov 18;18:eRC4641. doi: 10.31744/einstein_journal/2020RC4641 (PMC7687917; doi:10.31744/einstein_journal/2020RC4641)
Supplement: Translation

# Translation: Episódios de vômitos em recém-nascidos como consequência de membrana duodenal: relatos de dois casos

**Authors:** Gabriela Bonente Herculano de Andrade, Barbara Said Marin, Daniela Nasu Monteiro Medeiros, Mauricio Gustavo Ieiri Yamanari, Eduardo Juan Troster

**Affiliations:** 1 São PauloSP Brasil Hospital Israelita Albert Einstein, São Paulo, SP, Brasil.; 1 São PauloSP Brasil Faculdade Israelita de Ciências da Saúde Albert Einstein, Hospital Israelita Albert Einstein, São Paulo, SP, Brasil.

**Keywords:** Membrana duodenal, Refluxo gastroesofágico, Desnutrição, Crianças

## Abstract

Episódios de vômito em recém-nascidos são extremamente comuns e frequentemente atribuídos a refluxo gastresofágico. Os sintomas de vômito, no entanto, podem ser causados por outras complicações. Neste relato, apresentamos dois casos: um lactente masculino, com 1 mês de idade, e um feminino, com 2 meses, ambos apresentando episódios de vômitos que levaram à desnutrição. Alguns pediatras costumam atribuir o diagnóstico de refluxo gastresofágico a recém-nascidos que estão vomitando; mas parcela da população tem outras causas que levam a sintomas semelhantes. O pediatra deve estar atento aos sinais clínicos de perda de peso, desidratação e desnutrição, para investigar outras causas de vômitos.

## INTRODUÇÃO

Episódios de vômito em recém-nascidos são extremamente comuns e frequentemente atribuídos a refluxo gastresofágico (RGE), um processo fisiológico causado pela passagem do conteúdo gástrico para dentro do esôfago.^(^^1^^)^O vômito, no entanto, é esporádico e pode ser causado por outras complicações. A maioria dos erros inatos do metabolismo aparecem precocemente no período neonatal, com vômito e baixo ganho de peso.^(^^2^^)^

É importante avaliar quando um caso de vômito tem impacto significativo sobre a saúde e o estado nutricional do recém-nascido. A ocorrência de vômitos com perda de peso rápida, nas primeiras semanas de vida, deve levar à hipótese de anormalidade anatômica do trato digestivo. Polidrâmnio no pré-natal, detectado na ultrassonografia morfológica, pode ser indicativo de obstrução intestinal fetal.^(^^1^^)^

Neste relato, os autores trazem os casos de um menino de 1 mês de idade e de uma menina de 2 meses. Ambos apresentavam episódios de vômitos que levaram à desnutrição. Inicialmente, pensou-se que poderia ser RGE. Após exame mais detalhado, o diagnóstico diferencial mostrou membrana duodenal. O caso do menino necessitou intervenção cirúrgica.

## RELATO DE CASO

### Caso 1

Um lactente do sexo masculino, com 1 mês de idade, foi levado à nossa instituição devido a vômitos recorrentes desde o nascimento e desnutrição aparente. O paciente aparentava mal estado geral, com muito baixo peso. Vômito bilioso era o único sintoma, mas o choro indicava dor intensa.

Alguns dias antes, o paciente estivera em outro hospital, onde foram relatados perda de peso excessiva e os episódios de vômito. Inicialmente, o diagnóstico foi RGE. Foi decidido que ele seria amamentado com mais frequência, complementando com fórmula, porém os vômitos aumentaram. O abdômen estava distendido e sensível à palpação. Estenose hipertrófica de piloro foi sugerida como diagnóstico diferencial.

Foi realizada ultrassonografia (US) abdominal em nosso hospital ( Figuras 1 a 3 ), a qual excluiu estenose pilórica ou outras lesões no abdômen. Foi detectada membrana duodenal: havia uma obstrução na segunda porção do duodeno com dilatação em direção cefálica.

**Figura 1:** Ultrassonografia com transdutor linear de frequência evidenciando distensão do antro gástrico (A) e bulbo duodenal (B). A membrana duodenal reduz o lúmen da segunda porção do duodeno (setas)

**Figura 2:** Ultrassonografia com transdutor linear de frequência evidenciando distensão do bulbo duodenal, (B) com afunilamento abrupto na segunda porção do duodeno (setas), correspondendo ao local da membrana duodenal

**Figura 3:** Ultrassonografia com transdutor linear de frequência evidenciando distensão do a bulbo duodenal com afunilamento abrupto na segunda porção do duodeno (setas), correspondendo ao local da membrana duodenal

A criança estava intensamente desnutrida e foi internada na unidade de terapia intensiva (UTI). A seguir, foi submetida à cirurgia abdominal. Foi realizada excisão total da membrana. O pós-operatório transcorreu sem intercorrências, exceto candidíase perineal causada pelos antibióticos. A criança recebeu alta no 20º dia de pós-operatório. Permanecia bem após 1 ano de seguimento.

### Caso 2

Lactente do sexo feminino, com 2 meses, internada na UTI pediátrica do Hospital Municipal Dr. Moysés Deutsch, em São Paulo (SP), Brasil durante 28 dias, com história de nascimento a termo, Apgar 9/10, peso ao nascer 3.260g e sem complicações durante a gestação. Os relatos eram de dificuldade para mamar e vômitos biliosos frequentes desde o nascimento, baixo ganho de peso e tendo apresentado icterícia neonatal (bilirrubina total de 17) com fototerapia por 1 dia. Como a perda de peso progrediu, e os episódios de vômito pioraram, foi admitida no hospital, apresentando desidratação e exames laboratoriais que mostraram alcalose metabólica hipoclorêmica e hiponatremia. Após a estabilização clínica, foi feita cirurgia pediátrica por laparotomia exploratória, com diagnóstico realizado durante a cirurgia. Os achados foram: bandas duodenais de Ladd, rotação intestinal e membrana duodenal obstruindo a luz do órgão. Esses achados não foram observados no ultrassom. Na cirurgia, foram realizadas lise das bandas de Ladd, anastomose duodeno-duodenal e apendicectomia tática devido a uma obstrução da segunda porção duodenal.

No pós-operatório, a bebê apresentou vômito e hipoglicemia persistente (dextro e glicose sérica diferindo no momento da hipoglicemia), necessitando de altas concentrações de glicose e hidrocortisona (25mg/^2^). No terceiro e no oitavo dias pós-operatórios, a paciente apresentou evisceração, com necessidade de reintrodução das alças intestinais. Com a piora do quadro clínico, a antibioticoterapia (vancomicina e meropenen) foi prorrogada. No 11º dia de pós-operatório, evoluiu para choque séptico por fungo (com crescimento de *Candida albicans* em hemocultura de cateter central de inserção periférica - PICC), necessitando de drogas vasoativas e ventilação mecânica. Dois dias depois, a condição da paciente melhorou, e ela foi extubada. Recebeu transfusão de concentrado de hemácias e evoluiu com edema palpebral, crepitação e dispneia, necessitando de reintubação em função de piora bilateral das imagens radiológicas, tendo lesão pulmonar aguda associada à transfusão (TRALI) como causa provável.

A paciente teve alta após 3 meses de hospitalização.

## DISCUSSÃO

A obstrução duodenal congênita é uma causa comum de obstrução intestinal em recém-nascidos.^(^^3^^)^Esse defeito é causado por uma alteração na canalização do duodeno que ocorre entre a oitava e a décima semanas de gestação. A obstrução duodenal congênita ocorre em cerca de um em 2.500 a 10 mil nascidos vivos (quase metade de todos os casos de obstrução intestinal neonatal). Outros estudos mostram que a incidência é cerca de um em 5.000 a 10 mil nascidos vivos, e quase 50% são associados a outras malformações.^(^^4^^-^^6^^)^Os diagnósticos diferenciais para vômitos em recém-nascidos são infecção, malformações e doenças metabólicas.

A taxa de mortalidade foi de 6% a 58%, mas, em estudos recentes, a taxa de sobrevida pós-operatória inicial melhorou de 60% para 90%. Mais de 50% das crianças têm outras anomalias, como cardiopatias (20%), má rotação (20%), síndrome de Down (30%) e outras. A sobrevida dos neonatos melhorou devido aos avanços em manejo cirúrgico, medicina intensiva e suporte nutricional pós-operatório.^(^^3^^,^^7^^)^Nosso paciente do sexo masculino não apresentava outras malformações.

Essas obstruções podem ser classificadas como completas ou parciais, e as causas são classificadas como intrínsecas (atresia e membrana duodenal) ou extrínsecas (má rotação com banda de Ladd e pâncreas anular). Também são classificadas de acordo com os tipos de membrana duodenal: membrana intacta que pode causar obstrução total e membrana perfurada que leva à obstrução parcial (2% dos casos). A membrana geralmente está localizada na segunda porção do duodeno, próxima à ampola de Vater.^(^^7^^-^^9^^)^

Há muitas manifestações clínicas relacionadas ao tipo de obstrução, como náuseas, vômitos e perda de peso. Quando a criança tem uma obstrução parcial, é difícil fazer o diagnóstico. No caso do paciente do sexo masculino, as fezes não tinham acolia, sugerindo obstrução parcial. Quando o diagnóstico é tardio, o paciente pode evoluir com desnutrição, desidratação e desequilíbrio hidroeletrolítico. Um estudo mostrou como apresentações mais comuns, vômito (100% dos casos – não bilioso em 19% e bilioso em 81%), distensão epigástrica em 70% e desidratação em 22% dos neonatos. Em casos graves, choque está presente em quase 19% das crianças. Este estudo mostrou que 37% dos recém-nascidos apresentam sintomas após 10 dias de vida.^(^^7^^-^^9^^)^

Existe a possibilidade de diagnóstico pré-natal, e a intervenção endoscópica poderia ser uma opção.^(^^8^^)^

Os pediatras devem considerar os diagnósticos diferenciais em caso de vômitos, principalmente quando o paciente não ganha peso, como nestes casos.

## CONCLUSÃO

Alguns pediatras com frequência atribuem o diagnóstico de refluxo gastresofágico a recém-nascidos com vômitos; no entanto, há uma porcentagem dessa população que tem outras causas que levam a sintomas semelhantes. Os pediatras devem estar alertas aos sinais clínicos de perda de peso, desidratação e desnutrição para investigar outras causas de vômitos.
